# Eight versus 28-point lung ultrasonography in moderate acute heart failure: a prospective comparative study

**DOI:** 10.1007/s11739-022-02943-9

**Published:** 2022-02-18

**Authors:** Antonio Leidi, Guillaume Soret, Tamara Mann, Flora Koegler, Matteo Coen, Alexandre Leszek, Laetitia Dubouchet, Alexandre Guillermin, Myriam Kaddour, Frédéric Rouyer, Christophe Combescure, Sebastian Carballo, Jean-Luc Reny, Christophe Marti, Jérôme Stirnemann, Olivier Grosgurin

**Affiliations:** 1 General Internal Medicine, Department of Medicine, Geneva University Hospitals, Rue Gabrielle-Perret-Gentil 4, 1205 Geneva, Switzerland; 2 Unit of Development and Research in Medical Education (UDREM), Faculty of Medicine, University of Geneva, Geneva, Switzerland; 3 Emergency Medicine, Department of Acute Medicine, Geneva University Hospitals, Geneva, Switzerland; 4 Clinical Research Center and Division of Clinical Epidemiology, Department of Health and Community Medicine, University of Geneva and Geneva University Hospitals, Geneva, Switzerland

**Keywords:** Lung ultrasonography, Ultrasound, Heart failure, Congestion, Protocol, Eight

## Abstract

Lung ultrasonography (LUS) is an accurate method of estimating lung congestion but there is ongoing debate on the optimal number of scanning points. The aim of the present study was to compare the reproducibility (i.e. interobserver agreement) and the feasibility (i.e. time consumption) of the two most practiced protocols in patients hospitalized for acute heart failure (AHF). This prospective trial compared 8- and 28-point LUS protocols. Both were performed by an expert–novice pair of sonographers at admission and after 4 to 6 days on patients admitted for AHF. A structured bio-clinical evaluation was simultaneously carried out by the treating physician. The primary outcome was expert-novice interobserver agreement estimated by kappa statistics. Secondary outcomes included time spent on image acquisition and interpretation. During the study period, 43 patients underwent a total of 319 LUS exams. Expert–novice interobserver agreement was moderate at admission and substantial at follow-up for 8-point protocol (weighted kappa of 0.54 and 0.62, respectively) with no significant difference for 28-point protocol (weighted kappa of 0.51 and 0.41; *P* value for comparison 0.74 at admission and 0.13 at follow-up). The 8-point protocol required significantly less time for image acquisition at admission (mean time difference − 3.6 min for experts, − 5.1 min for novices) and interpretation (− 6.0 min for experts and − 6.3 min for novices; *P* value < 0.001 for all time comparisons). Similar differences were observed at follow-up. In conclusion, an 8-point LUS protocol was shown to be timesaving with similar reproducibility when compared with a 28-point protocol. It should be preferred for evaluating lung congestion in AHF inpatients.

**Supplementary Information:**

The online version contains supplementary material available at 10.1007/s11739-022-02943-9.

## Introduction

Despite therapeutic advances, acute heart failure (AHF) remains the leading cause of hospital admission and one of the most frequent reasons for readmission in northern countries [1–3]. As the main reason for AHF hospitalization is congestion-driven symptoms, the cornerstone of treatment is decongestive therapy [4]. In the absence of specific quantitative measures, however, residual congestion is noted at discharge in 10–15% of patients and is associated with an increased risk of readmission [5]. Lung ultrasonography (LUS) has been progressively incorporated into medical practice [6]. LUS has high level of accuracy for extravascular lung water detection (EVLW) and provides a semi-quantification of congestion, even at subclinical stage [7, 8]. Decrease in B-lines, the sonographic hallmark of cardiogenic edema, correlates with clinical improvement and can be used to guide decongestion [9–12], whereas their persistence after treatment is associated with an increased risk of hospital admission [13–15].

The several existing protocols differ in exhaustiveness (i.e. number and localization of scanning points) and rating methodology [16]. Eight- and 28-point protocols are generally preferred when following patients suffering from heart failure [17]. Eight-point protocols seem to have similar diagnostic value but less time when performed at admission in emergency departments (ED) or intensive care units (ICU) [18, 19]. No comparative data exist for less congested patients, such as AHF inpatients. A short training period is sufficient to recognize B-lines; indeed the learning curve is known to be sharper than for other US techniques [20, 21]. Nevertheless, in most studies only experienced sonographers performed and interpreted LUS, raising the question of generalizability of results.

The aim of the present study was to compare 8- and 28-point LUS protocols in terms of reproducibility (expert-novice interobserver agreement), feasibility (time for images acquisition and interpretation), and performance (correlation with clinical features and biomarkers).

## Methods

The present article was written in accordance with the ESC reporting checklist for lung ultrasound studies in heart failure cohorts [22], the STROBE Statement checklist and was registered in clinicaltrial.gov (NCT 04,174,794). The investigation conforms with the principles outlined in the Declaration of Helsinki and was approved by the local ethics committee (CCER 2019–01,596). Informed consent was obtained from all patients prior to inclusion.

This single-center prospective observational study included adults hospitalized consecutively for AHF regardless of left ventricular ejection fraction. AHF was defined according to ESC criteria [4] (presence of ≥ 1 sign or symptom and a value of N-terminal-pro-B-type natriuretic peptide (NT-proBNP) of ≥ 300 ng/l). Participants were included when both expert and novice sonographers were available. Patients admitted directly to ICU were excluded in addition to those with comorbidities known to produce B-line artefacts (i.e. interstitial lung diseases, ARDS, lung cancer or metastasis, lung contusion, previous lung surgery). Patients with oligo-anuric end stage kidney disease and unwillingness or inability to give consent were also excluded. To avoid unnecessary patient selection, a concomitant diagnosis of pneumonia was not considered an exclusion criterion, even if this condition can present with B-lines. Setting, recruitment and procedures are detailed in Appendix 1, 2.

### Lung ultrasonography

All images were obtained with high-end devices; details on knobology and ultrasonography procedures are described in Appendix 3.

### Eight-point protocol

**Fig. 1:**
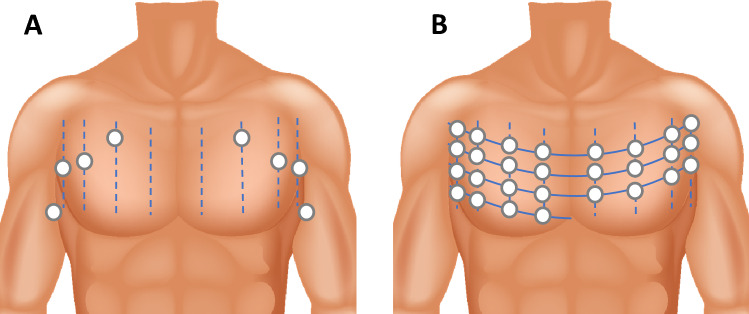
Lung ultrasonography protocols. In eight-point protocol (Panel **A**) thorax is explored bilaterally in second intercostal space (ics) on mid-clavicular line (mcl), in forth ics on anterior axillary line (aal), in fifth ics on mid-axillary line (mal) and in the seventh ics beyond the posterior axillary line. In twenty-eight-point protocol (Panel **B**) thorax is explored from the second to the fifth ics in right hemithorax and from the second to the fourth ics in left hemithorax, along four thoracic lines (parasternal line, mcl, aal, mal)

This protocol was adapted from existing protocols [23, 24] and is represented in Fig. 1. The transducer was oriented in a sagittal plan to visualize one ICS and two ribs with their shadows. A 1 centimetre lateral translation of the probe in each direction was allowed to obtain a better acoustic window. Every point was coded (*p*= 1) in presence of ≥ 3 B-lines simultaneously on a frozen image or in presence of pleural effusion. This was introduced as we considered pleural effusion as a marker of congestion. The total score ranged from 0 to 8.

### Twenty-eight-point protocol

In the 28-point protocol the thorax was scanned from the second to the fifth ICS in right hemithorax and from the second to the fourth ICS in left hemithorax, following four thoracic lines (Fig. 1). The sum of the maximum number of B-lines visualized on a frozen image for each scanning point yielded a score denoting the extent of the pulmonary congestion. According to the original description [25], the transducer was oriented in a transversal plan allowing a larger visualization of the pleural line. When visualization of B-lines was impeded by extra-pulmonary structures (e.g. heart) or pleural effusion, the affected point scored zero B-lines.

### Outcomes

The primary outcome was interobserver agreement between expert and novice sonographers for both protocols. To compare protocols using different grading systems, results were rated with a common pre-specified 4-levels lung congestion scale (LCS). For the 28-point protocol, lung congestion was classified in accordance with the literature as severe (> 30), moderate (16–30), mild (6–15) or absent (≤ 5 B-lines). The 8-point protocol was arbitrarily predefined as follows: severe (6–8), moderate (4–5), mild (2–3) and absent (0–1 positive points). To emphasize full decongestion, both LCS were additionally categorised in a dichotomous way (i.e. absence/presence of congestion) using a cut-off of ≤ 5 B-lines and ≤ 1 positive points for the 28-point and 8-point protocols, respectively. As secondary outcomes, we measured the time spent for image acquisition and for interpretation. Additionally, change in B-lines after decongestive therapy was analyzed by computing admission-follow-up difference in LCS. It was subsequently correlated with the EVEREST score, body weight and NT-proBNP evolution. Finally, we explored the relationship between aLUS and length of stay as well as fLUS and short-term readmission and mortality.

### Potential sources of bias

Sonographer competence, patient body mass index (BMI), time since diuretic administration, patient position, ultrasound device, knobology and image processing could potentially impact the B-lines count. To limit the influence of part of these variables, LUS scans were executed within a 60 min timespan by both expert and novice, the patient lying in a pre-determined position (see Appendix 3). In addition, standardized US device, probe and image processing were used. Moreover, the primary outcome was estimated post-hoc in a sub-group of obese patients (BMI ≥ 30 kg/m^2^).

### Statistical analysis

In this exploratory study, a sample of 90 patients was initially planned to obtain a precision in estimate of kappa statistic around ± 0.12. However, due to recruitment suspension in March, 2020 in non-SARS-CoV-2 related studies due to cross-infection risks, 43 patients were in fact recruited. Characteristics of participants are presented with descriptive statistics with median and interquartile range for continuous variables and percentages for categorical variables. Expert–novice interobserver agreement was estimated by kappa statistics, with Cicchetti–Allison’s weighting. Differences in agreement between 8- and 28-point protocols were assessed independently at admission and follow-up using a permutation test. For US image acquisition and interpretation time differences, outcome comparison was conducted by paired *t* test. Length of stay and early readmission and mortality were compared with Wilcoxon rank test and Fisher’s exact test, respectively. A 2-sided *p* value of < 0.05 has been considered to infer statistical significance. Spearman correlation coefficient was used to assess correlation between evolution in LUS scores and bio-clinical variables between admission and follow-up; NT-proBNP delta was expressed in percentage. A post-hoc analysis was performed to assess correlation between LUS and bio-clinical congestion markers at admission and follow-up, separately. No replacement of missing data was planned.

## Results

**Fig. 2:**
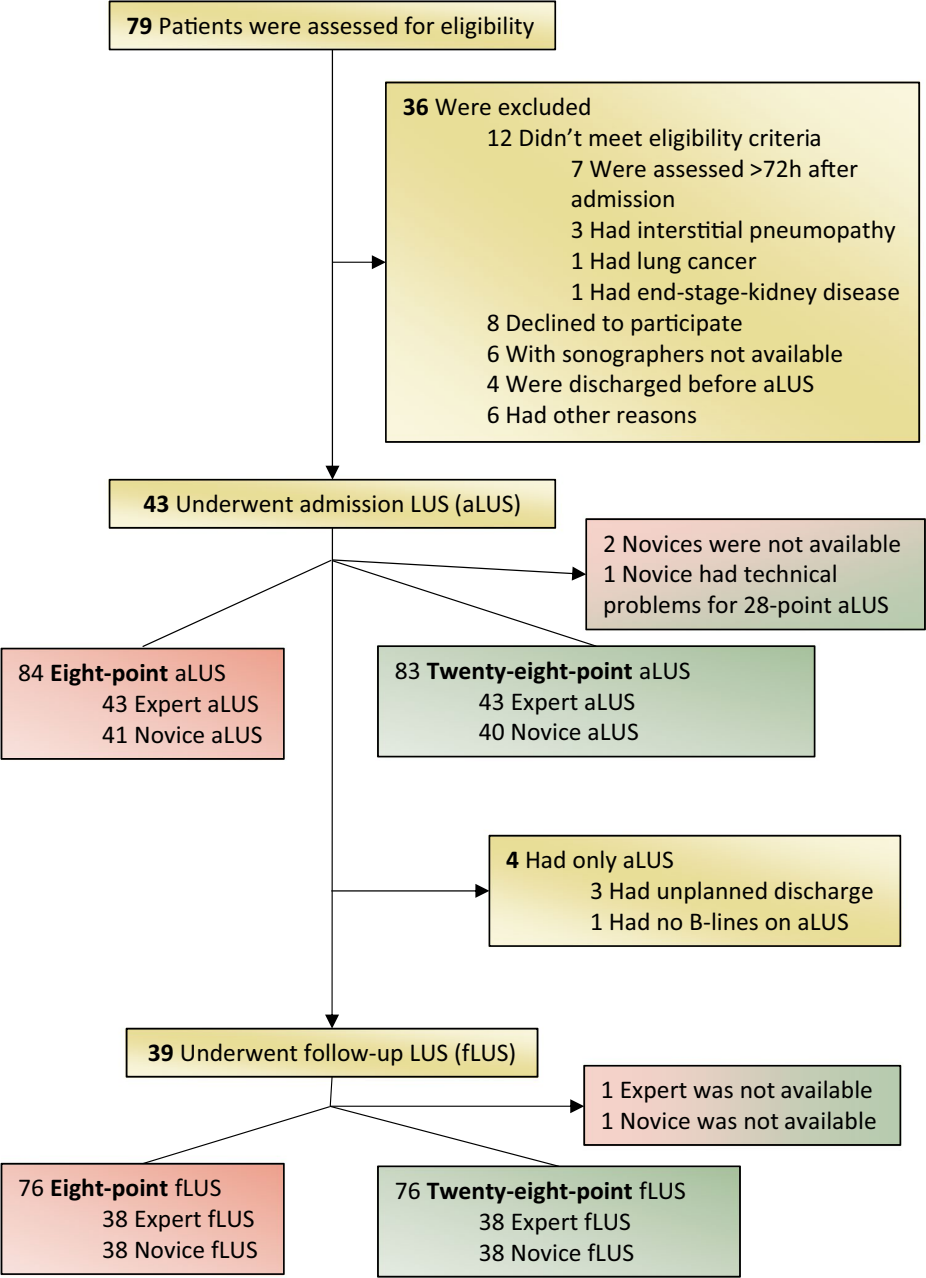
Study flow diagram

Between October 8th, 2019 and March 16th, 2020, 43 patients (mean age of 76 years, 26% of women, mean left ventricular ejection fraction of 43%) underwent up to 8 LUS exams for a total of 319, 162 performed by three expert and 157 by ten novice sonographers. All subjects had at least one aLUS and four (9%) had no fLUS due to unplanned early hospital discharge or the absence of B-lines on aLUS (Fig. 2). For approximately half of the patients this was their first hospitalization for heart failure and less than half of all patients had ejection fraction < 40% (Table 1). At inclusion, almost all patients (93%) presented signs of peripheral congestion (i.e. lower limbs oedema or lung rales) on physical examination but 20% showed no signs of pulmonary congestion on auscultation (Table 2).

**Table 1:** Baseline demographics and clinical characteristics of patients

	Total (*n* = 43)
Age, median (IQR), years	76 (65–84)
Men, *n* (%)	32 (74)
BMI, median (IQR), kg/m^2^	27 (24–32)
Medical history	
Prior heart failure, *n* (%)	23 (54)
Hypertension, *n* (%)	31 (72)
Diabetes mellitus, *n* (%)	18 (42)
Atrial fibrillation/flutter, *n* (%)	20 (47)
Valvulopathy, *n* (%)	23 (54)
CAD, *n* (%)	16 (37)
Chronic kidney disease, *n* (%)	12 (28)
Concomitant pneumonia, *n* (%)	5 (12)
Echocardiography	
LVEF, median (IQR), %	43 (27–60)
≥ 50%, *n* (%)	19 (44)
40–49%, *n* (%)	6 (14)
< 40%, *n* (%)	18 (42)
Serum creatinine, median (IQR), µmol/L	109 (87–142)
Serum hemoglobin, median (IQR), g/L	131 (104–145)
Serum albumin, median (IQR), g/L	37 (35–40)
Destination after discharge, *n* (%)	
Home	33 (77)
Rehabilitation	8 (19)
Other	2 (5)

*BMI* means body mass index, *LVEF* means left ventricular ejection fraction, *CAD* means coronary artery disease

**Table 2:** Key clinical and biological features of patients at admission and follow-up

	*N *= 43	Admission	*N* = 39	Follow-up
EVEREST score (total), median (IQR)	39	8 (5–10)	38	3 (2–6)
Dyspnea, *n* (%)	41		39	
None		5 (12)		22 (57)
Seldom		12 (29)		11 (28)
Frequent		17 (42)		6 (15)
Continuous		7 (17)		0 (0)
Orthopnea, *n* (%)	41		39	
None		17 (42)		20 (72)
Seldom		11 (27)		10 (26)
Frequent		7 (17)		1 (2)
Continuous		6 (14)		0 (0)
Fatigue, *n* (%)	41		39	
None		5 (12)		9 (23)
Seldom		9 (22)		23 (59)
Frequent		21 (51)		5 (13)
Continuous		6 (15)		2 (5)
JVD, *n* (%)	39		38	
≤ 6 cm H2O		20 (51)		26 (68)
6–9		11 (28)		9 (24)
10–15		7 (18)		3 (8)
≥ 15		1 (3)		0 (0)
Rales, *n* (%)	41		39	
None		8 (20)		19 (49)
Bases		25 (61)		18 (46)
Up to < 50%		5 (12)		2 (5)
≥ 50%		3 (7)		0 (0)
Oedema, *n* (%)	41		39	
Absent/trace		9 (22)		22 (56)
Slight		11 (27)		11 (28)
Moderate		9 (22)		3 (8)
Marked		12 (29)		3 (8)
Body weight, median (IQR), kg	42	82.8 (75.1–93.0)	41	78.8 (72.3–92.6)
Serum NT-proBNP, median (IQR), ng/L	43	4618 (1775–8066)	41	2587 (901–4295)

*JVD* means jugular vein distention

Imaging was 100% feasible for the 8-point protocol. In contrast, when performing the 28-point protocol, examination was impeded in 18% of scanning points by extra-pulmonary structures (e.g. abdominal organs, pleural effusion, pace-makers). Admission LUS were performed on average 1 day (IQR 1 to 3) after admission to the ward. Significant pulmonary congestion was detected by experts at admission in 86 and 91% of subjects using the 8-point and 28-point protocols, respectively, whereas pleural effusion was present in 72% of subjects. Proportions were lower for novices (67%, 91%, 50%, respectively). Follow-up LUS was performed after a median period of 4.5 days (IQR 4 to 6). Only one patient had delayed fLUS (14 days) due to a rapid decline in clinical condition requiring ICU admission. For all protocols, scores decreased at fLUS: 20 and 28% relative decrease in LCS was observed using 8-point protocol, 25 and 13% using 28-point protocol, for expert and novices, respectively (Appendix 4). Globally, congestion was more prevalent in lateral (particularly infero-lateral) than anterior zones (Appendix 5). For five patients a concomitant diagnosis of pneumonia was documented by treating physicians. Two patients needed unblinding and the communication of the expert aLUS results to the treating physician due to pre-specified potential life-threatening conditions as follows: absence of B-lines in a hypoxemic patient (potentially signalling the presence of pulmonary embolism) and presence of asymmetric isolated lung consolidation (compatible with pneumonia). Discharge diagnoses were right heart failure and pneumonia, respectively.

Overall, the median length of stay was 13 days (IQR 5 to 17) with most patients being discharged home (77%). Cumulative mortality and readmission rate at day 30 post-discharge was 16% (2 deaths and 5 readmissions). Proportions were higher at day 60 post-discharge (Total 23%, mortality 5%, readmissions 18%).

### Primary outcome

Expert–novice interobserver agreement was moderate at admission for both the 8-point (weighted kappa 0.54, 95% CI 0.35 to 0.74) and the 28-point protocol (0.51, 95% CI 0.31 to 0.71). Substantial interobserver agreement was obtained for the 8-point protocol at follow-up (0.62, 95% CI 0.47 to 0.77), whereas it was moderate for the 28-point protocol (0.41, 95% CI 0.25 to 0.57). However, the difference was not statistically significant (*P* = 0.74 at admission and *P* = 0.13 at follow-up). Results did not substantially differ in a subgroup of patients with BMI ≥ 30 kg/m^2^ (Table 3) nor were they influenced by the increased experience of novice sonographers throughout the study (Appendix 6). Bland–Altman plots are available in Appendix 7.

**Table 3:** Interobserver agreement at admission and follow-up in all patients and according to body mass index

	Weighted kappa (95% CI)	
	Admission (aLUS)	Follow-up (fLUS)
All patients (*n* = 43)		
8-point protocol	0.54 (0.35–0.74)	0.62 (0.47–0.77)
28-point protocol	0.51 (0.31–0.71)	0.41 (0.25–0.57)
*P* value for comparison	0.74	0.13
Non obese patients (*n* = 25)		
8-point protocol	0.61 (0.37–0.86)	0.62 (0.43–0.81)
28-point protocol	0.59 (0.36–0.82)	0.34 (0.10–0.58)
Obese patients (*n* = 18)		
8-point protocol	0.43 (0.11–0.75)	0.57 (0.36–0.77)
28-point protocol	0.38 (0.04–0.72)	0.43 (0.22–0.64)

### Secondary outcomes

Image acquisition and interpretation time was significantly lower for the 8-point compared to 28-point protocol (*P* < 0.001 for all comparisons). On average, the 8-point protocol required less than 3 min for experts (aLUS: 2.95 min; fLUS: 2.8 min) compared with more than the double that time for the 28-point (aLUS: 6.52 min; fLUS: 6.23 min); time difference − 3.6 min (95% CI − 4.2 to − 3.0) and − 3.4 (95% CI − 4.1 to − 2.8) at admission and follow-up, respectively. Novices spent more than 4 min performing the 8-point (aLUS: 4.12 min; fLUS: 4.7 min), and at least 9 min for the 28-point protocol (aLUS: 9.32 min; fLUS: 9.02 min); time difference − 5.1 min (95% CI − 5.9 to − 4.3) and − 4.3 min (95% CI − 5.1 to − 3.6). Mean times for acquisition and interpretation and between-protocol time differences are shown in Appendix 8. We found no significant correlation between expert LUS congestion score evolution and temporal change in NT-proBNP (*ρ* = 0.16, *P* = 0.37 for 8-point, *ρ* = 0.28, *P* = 0.09 for 28-point), body weight (*ρ* = 0.22, *P* = 0.18 for 8-point, *ρ* = − 0.21, *P* = 0.20 for 28-point) or EVEREST score (*ρ* = 0.15, *P* = 0.37 for 8-point, *ρ *= − 0.11, *P* = 0.53 for 28-point). Results and dot plots are presented in Appendix 9. Modest albeit significant correlation was observed between LUS and NT-proBNP values, when analysed separately at admission and at follow-up (Appendix 10, 11).

Interestingly, the length of hospital stay seems to be lower in 6 patients with no detectable congestion on expert 8-point aLUS (i.e. < 2/8 positive points: median 4.5 days, IQR 4 to 5) when compared to 37 patients with mild to severe congestion (i.e. ≥ 2/8 positive points: median 13 days, IQR 7 to 19, *P* = 0.015). Additionally, a trend to lower rates of 30- and 60 day readmission and mortality was observed in patients without congestion on expert 8-point fLUS as presented in Table 4. Results were similar when using expert 28-point LUS.

**Table 4:** In-hospital length of stay and early clinical outcomes according to lung ultrasonography

Admission expert LUS	Total	8-point protocol		*P* value	28-point protocol		*P* value
Degree of congestion	*n* = 43	Absent (*n* = 6)	Mild to Severe (*n* = 37)		Absent (*n* = 4)	Mild to Severe (*n* = 39)	
Length of stay, days, median (IQR)	13.0 (5.0–17.0)	4.5 (4.0–5.0)	13.0 (7.0–19.0)	0.02	8.0 (3.5–12.5)	13.0 (6.0–19.0)	0.21
Follow-up expert LUS							
Degree of congestion	*N* = 38^a^	Absent (*n* = 13)	Mild to severe (*n* = 25)		Absent (*n* = 11)	Mild to severe (*n* = 27)	
30-day readmission and mortality, *n* (%)	6 (16)	0 (0)	6 (24)	0.08	1 (9)	5 (18)	0.65
60-day readmission and mortality, *n* (%)	9 (24)	1 (8)	8 (32)	0.13	2 (18)	7 (26)	1.00

^a^one patient died during the index hospitalisation

## Discussion

In this prospective comparative study, pulmonary congestion was detected by LUS in the majority of patients at admission and decreased at follow-up. Whereas significant congestion was detected in a greater proportion of patients by both experts and novices when using 28-point protocol, the 8-point protocol required significantly less time for imaging and interpretation. A previous study of 20 ICU patients showed a reduction in examination time with no significant reduction in B-lines detection when decreasing the number of scanning points from 28 to 8 or 6 [18]. In another recent multicentric study, the diagnostic value of several LUS protocols were compared in dyspnoeic ED patients. One-hundred-seventeen subjects underwent the 28-point protocol at admission. Four, 6- and 8-point protocols were derived post hoc by selecting part of the 28 recorded video clips. The eight-point protocol was associated with a significant increase in diagnostic accuracy in a subset of patients with an uncertain diagnosis following clinical assessment [19]. In this trial, however, results are exposed to bias due to protocols not being performed independently. Moreover, derivation of 8 from 28-point protocol prevented sonographers from exploring posteriorly to the mid-axillary line, where EVLW tends to cumulate in a semi-recumbent patients as shown in our study (Appendix 5) and in previous reports [10]. These results may, therefore, not be applicable in less congested subjects as in hospitalized AHF patients.

In both cited studies, only trained sonographers performed LUS. It is worth noting that, if interobserver agreement is generally considered substantial for LUS, most studies are based on post-hoc off-line review of video loops acquired by a unique expert sonographer [26]. Image acquisition could, however, be an important source of variability, particularly for pairs of expert–novice sonographers. In our study LUS was performed and interpreted real-time independently by both experts and novices and we observed moderate to substantial agreement with no significant difference between protocols. Our findings are concordant with a prior report of 91 ED dyspneic patients undergoing a 10-zones LUS performed bedside by pairs of expert–novice sonographers, observing moderate agreement in counting B-lines (ICC 0.59) [27].

Early publications claimed that a 28-point protocol required < 3 min [28]; in contrast to subsequent reports suggesting 5 to 15 min was nearer the case thus rendering it impractical for daily clinical practice, especially in emergency settings [16]. In the present study the 28-point protocol took an average of 6 and 9 min for experts and novices, respectively; scanning time was reduced by more than 50% with 8-point protocol. In clinical practice LUS is interpreted during acquisition. The separating of image acquisition and interpretation, due to the study design, may have artificially overestimated total time.

Despite 28-point LUS being feasible in all patients, one-fifth of scanned points was invalid due to visualization of extra-pulmonary structures. With the 8-point protocol, imaging was possible in 100% of scanning points. This and the fact that the same regions of thorax are explored may explain the limited loss of information when using reduced scanning point protocols.

This study showed modest albeit significant correlation between LUS and NT-proBNP values at admission and follow-up. No significant correlation, however, was highlighted between the decrease of LUS congestion and clinical evolution, weight loss and NT-proBNP decline, irrespective of the protocol used. Similarly, a previous study did not find significant correlation between admission-discharge delta BNP and delta LUS [10]. In contrast, in this study, delta LUS correlates significantly with delta clinical congestion score (*r *= 0.49*, P* < 0.05). When compared to this study, our patients had lower clinical congestion at admission (median value of 8/10 versus 8/18, respectively), and lower decrease at follow-up (− 89% versus − 63%), explaining differences in results.

Clinical appreciation of volemia is difficult, residual congestion at discharge is frequent and seems to be a key factor in hospital readmissions, even at subclinical stage [8]. In our study, rales were judged absent in one quarter of patients who still had significant LUS congestion at follow-up. Interestingly, patients with persistent congestion on expert 8-point fLUS (i.e. ≥ 2/8 positive points) had higher rate of post-discharge mortality and readmission at 30 days (24% versus 0%) and 60 days (32% versus 8%, Table 4) indicating the prognostic value of LUS congestion on early clinical outcomes, as previously shown in hospitalized and ambulatory heart failure patients [13, 29]. Interestingly, in a previous study 8- and 28-point LUS similarly predict clinical outcomes [30]. Complete LUS decongestion before discharge may, therefore, be a valuable target to improve early clinical outcomes. If recent studies suggest that an ambulatory LUS-driven decongestion strategy may reduce unplanned urgent visits or hospital admissions in chronic heart failure patients [11, 12, 31], no data are currently available for AHF inpatients.

This study has certain limitations. First, the collected sample for this exploratory study was modest, due to recruitment interruption during the COVD-19 pandemic, and the precision of kappa statistics was lower than planned, ranging from ± 0.2 at admission to ± 0.15 at follow-up, instead of the planned ± 0.12. Second, the 8-point protocol used in this study was not mentioned in the international guidelines on lung ultrasonography [17]. These guidelines have not been updated since 2012, whilst the 8-point protocol was introduced in the past decade [23, 24]. Third, the exclusion of severely congested AHF patients (i.e. requiring ICU admission) may affect generalizability in that population. However, benefits of LUS in AHF are more marked when pulmonary congestion is moderate, and its clinical detection becomes challenging. Additionally, interobserver concordance is more easily achieved for extremes (i.e. high and low number of B-lines) than for intermediate levels of congestion [27]. Finally, sonographers could not be blinded to patients and this may have influenced LUS interpretation.

## Conclusions

In spite of its limitations, the present study has succeeded in bringing two essential answers to the ongoing LUS protocol debate. There is moderate to substantial agreement between experts and novices after a short, structured training period, when LUS is executed and interpreted independently at the bedside. Further trials should, in our opinion, include novices amongst study sonographers. Moreover, in AHF inpatients we found no benefit in terms of reproducibility in using an exhaustive 28-point protocol which required more than double the time in image acquisition and interpretation. Future research and clinical efforts could be concentrated in LUS protocols with limited scanning points.

## Supplementary Information

Below is the link to the electronic supplementary material.

Supplementary file1 (DOCX 2937 KB)
