# Supplementary material for: Eight versus 28-point lung ultrasonography in moderate acute heart failure: a prospective comparative study
Source: Intern Emerg Med. 2022 Feb 18;17(5):1375–83. doi: 10.1007/s11739-022-02943-9 (PMC8856869; doi:10.1007/s11739-022-02943-9)
Supplement: Supplementary file 1 — Supplementary file1 (DOCX 2937 KB) [file 11739_2022_2943_MOESM1_ESM.docx]

**Eight versus Twenty-eight Point Lung Ultrasonography in Moderate Acute Heat Failure: a prospective comparative study**

**Appendices**

**Authors:**

Antonio Leidi MD, Guillaume Soret MD, Tamara Mann RN, Flora Koegler MD, Matteo Coen MD PhD, Alexandre Leszek MD, Laetitia Dubouchet MD, Alexandre Guillermin MD, Myriam Kaddour MD, Frédéric Rouyer MD, Christophe Combescure PhD, Sebastian Carballo MD PhD, Jean-Luc Reny MD PhD, Christophe Marti MD PD, Jérôme Stirnemann MD PhD, Olivier Grosgurin MD

**Corresponding author:**

Antonio Leidi, General Internal Medicine, Department of Medicine, Geneva University Hospitals, Rue Gabrielle-Perret-Gentil 4, 1205 Geneva, Switzerland, tel: +41795534559, fax: +41223729202, [Antonio.Leidi@hcuge.ch](mailto:Antonio.Leidi@hcuge.ch)

**Appendix 1. Setting, recruitment, procedures and data sources**

This study is part of a more comprehensive ongoing HF register that has been recruiting patients since 2014. Patients were recruited from the Geneva University Hospitals’ general internal medicine and cardiology inpatient units. All patients admitted to these units were routinely screened each working day by a study nurse for a diagnosis of AHF. Participation in the study was proposed to eligible patients within 72 hours of ward admission and informed consent obtained. Patients subsequently underwent both an 8 and 28-point LUS protocol by a pair of expert-novice sonographers. They independently performed the protocols in sequence and were reciprocally blinded. The eight-point protocol was systematically undertaken first to avoid potential influences from the more exhaustive 28-point protocol. In addition, sonographers were masked to clinical data files. Results of LUS were only to be communicated to the doctor in charge of the patient in case of pre-specified life-threatening condition detection (i.e. absence of pleural sliding, unilateral isolated lung consolidation, absence of alveolo-interstitial syndrome in a hypoxemic patient). Four to six days after admission LUS (aLUS) or the day of discharge if before the fourth day, the procedure was repeated by same or different sonographers and named follow-up LUS (fLUS). On the same days of LUS, the treating physician independently assessed clinical congestion, calculated and documented the EVEREST congestion score (1) along with body weight and measured NT-proBNP if no value was available in the preceding 24 hours (see Appendix 2). Immediately after carrying out each LUS protocol, sonographers interpreted the acquired images at the bedside visualizing the saved video loops. The 8-point protocol results were reported on a paper case report form before executing and interpreting the 28-point protocol. Time spent for image acquisition and interpretation was measured with a self-enabled chronometer. The treating physician was asked to complete a paper case report form containing the EVEREST score, body weight and NT-proBNP value on the same day (see Appendix 2). Paper case report forms were subsequently entered in the database with double checking by an independent data service provider (Data Conversion Service SA, Geneva, Switzerland). Baseline patient characteristics obtained from patients or inpatient clinical files were collected and documented by the study nurse. Following discharge, patients and/or general practitioners were contacted by phone to identify time to readmission or mortality.

**Appendix 2. Clinical and Echographic case report forms**


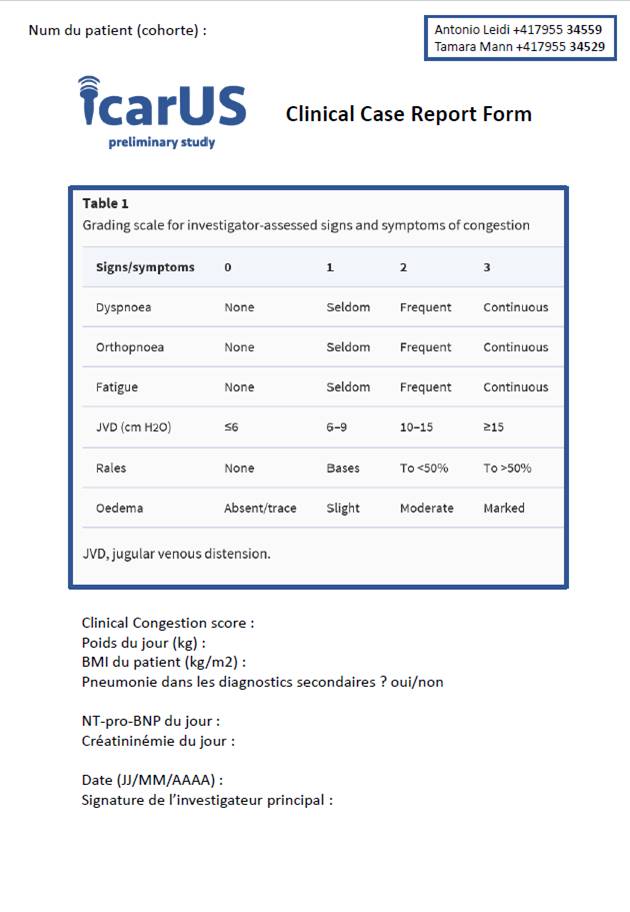


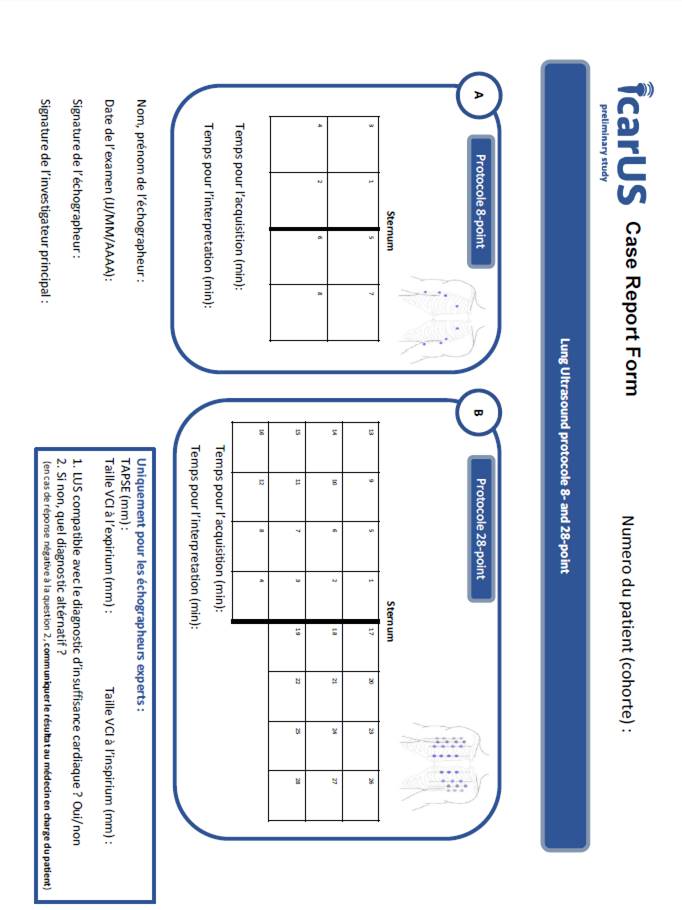


**Appendix 3. Lung ultrasonography**

Images were obtained with two Philips Sparq® ultrasound systems high-end devices (Philips AG Health Systems, Zurich, Switzerland). A phased array (cardiac) probe with pre-set depth of 15 centimetres and 60 percent of gain was used. Tissue harmonics were switched off. Four protocols were performed for each patient (two by experts and two by novices); to limit the duration of these procedures, video loops of 3 seconds were recorded for each scanning point. Sonographers were free to change knobology according to their judgement. Since B-lines are susceptible to vary with time after diuretic administration and patient position, both sonographers were required to complete LUS within a 60-minute time span, with the patient lying in a pre-specified 30-45° semi-recumbent position. Expert sonographers were defined as having performed >100 LUS prior to the study, as previously described (2). Novice sonographers attended a 90-minutes specific theoretical and practical training session and performed five supervised LUS on patients hospitalized for AHF (both protocols at least twice) but had limited or no previous experience in LUS. Both protocols explored the anterolateral thorax bilaterally.

**Appendix 4. Eight- and Twenty-eight-point protocol results at admission and follow-up**

|  | Expert | | Novice | |
| --- | --- | --- | --- | --- |
| **Eight-point protocol** | Admission | Follow-up | Admission | Follow-up |
| Total score, median (IQR) | **3.0 (2.0 to 4.0)** | **2.0 (1.0 to 4.0)** | **2.5 (0.0 to 4.0)** | **2.0 (0.0 to 3.0)** |
| 0-1, n (%) | 6 (14) | 13 (34) | 14 (33) | 15 (40) |
| 2-3, n (%) | 22 (51) | 15 (40) | 15 (36) | 18 (37) |
| 4-5, n (%) | 11 (26) | 7 (18) | 9 (21) | 3 (8) |
| 6-8, n (%) | 4 (9) | 3 (8) | 4 (10) | 2 (5) |
| Detection of pleural effusion, n (%) | 31 (72) | 24 (59) | 19 (50) | 19 (50) |
| **Twenty-eight-point protocol** |  |  |  |  |
| Total score, median (IQR) | **19.0 (11.0 to 27.0)** | **13.5 (5.0 to 24.0)** | **20.5 (10.0 to 32.0)** | **17.0 (9.0 to 28.0)** |
| 0-5, n (%) | 4 (9) | 11 (30) | 4 (9) | 4 (11) |
| 6-15, n (%) | 10 (23) | 10 (26) | 12 (29) | 13 (34) |
| 16-30, n (%) | 21 (49) | 12 (32) | 15 (36) | 13 (34) |
| >30, n (%) | 8 (19) | 5 (13) | 11 (26) | 8 (21) |
| **Delay from admission**, median (IQR), days | 1 (1 to 3) | - | 1 (1 to 3) | - |
| **Delay from aLUS**, median (IQR), days | - | 4.5 (4 to 6) | - | 4.5 (4 to 6) |

**Appendix 5. Eight-point protocol results by anatomical region at admission and follow-up**


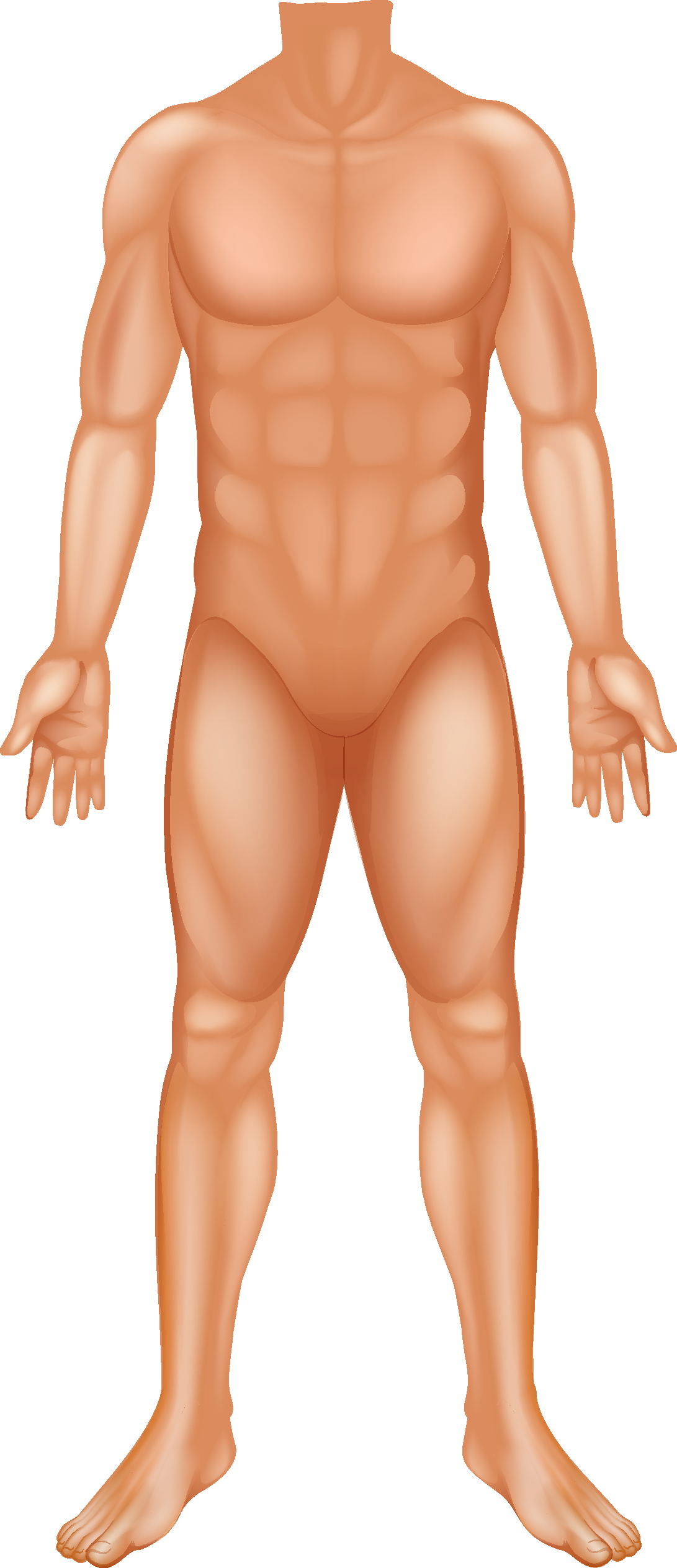

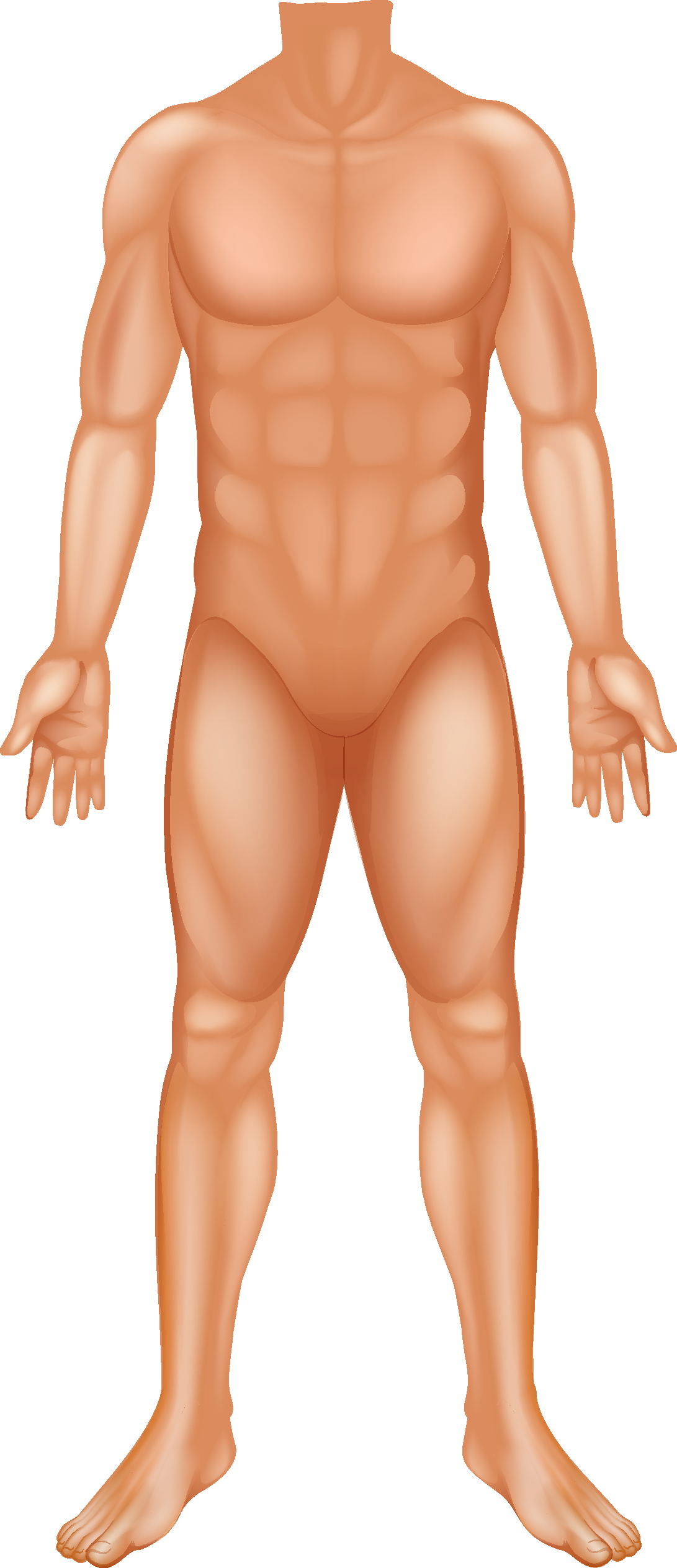

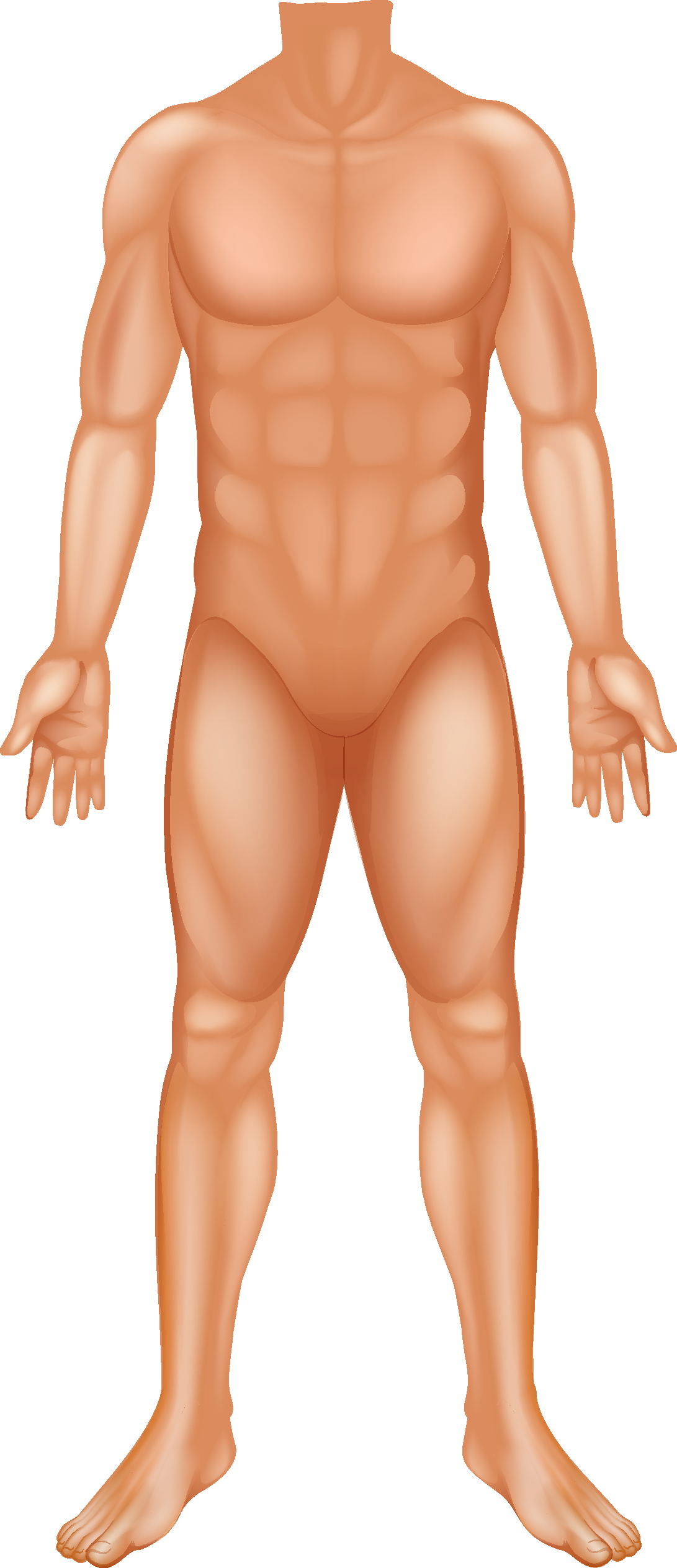

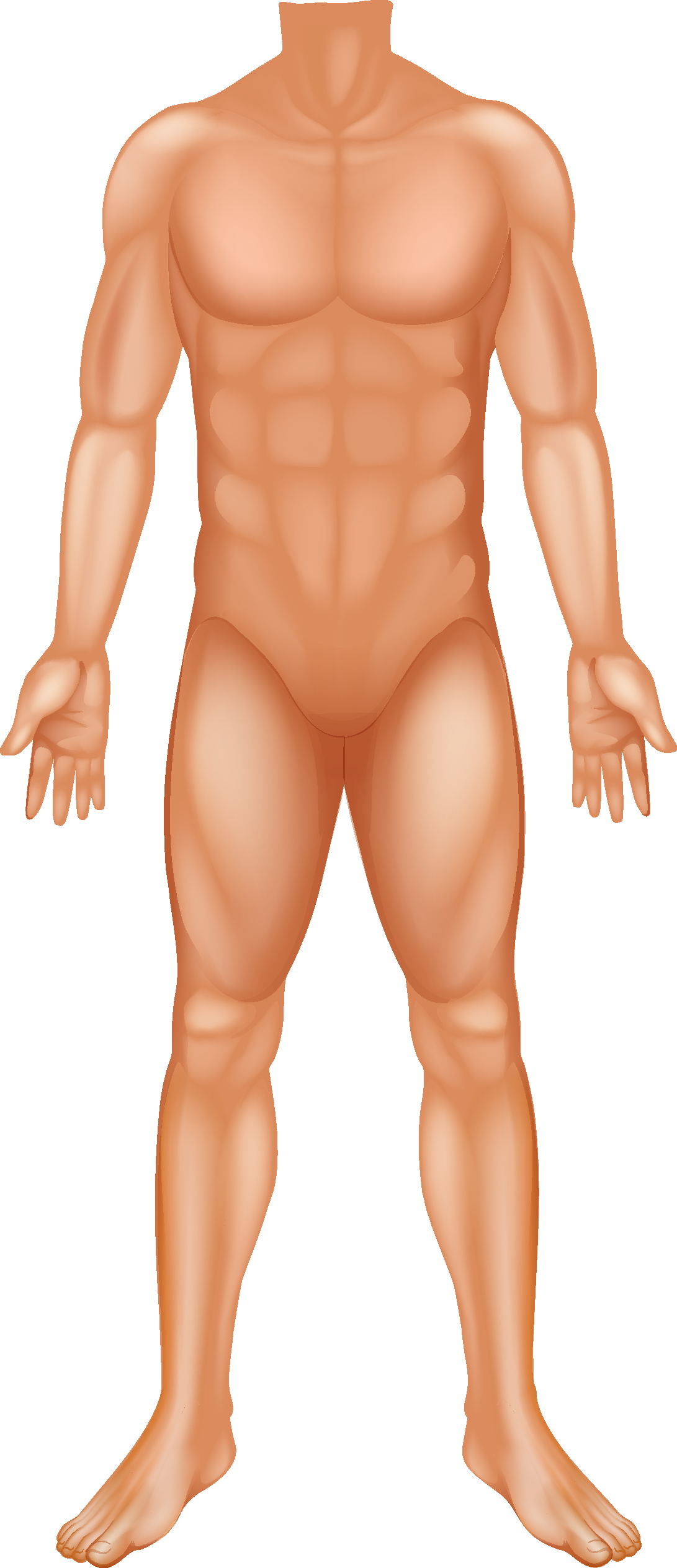


**8/43**

**8/43**

**18/43**

**38/43**

**9/43**

**10/43**

**11/43**

**34/43**

**5/38**

**7/38**

**11/38**

**25/38**

**5/38**

**7/38**

**10/38**

**26/38**

**8/41**

**9/41**

**16/41**

**27/41**

**4/41**

**7/41**

**11/41**

**27/41**

**4/38**

**6/38**

**11/38**

**19/38**

**4/38**

**5/38**

**7/38**

**19/38**

Panel A shows proportion of patient with positive point at admission (A1) and follow-up (A2) for expert sonographers. Same proportions are presented for novices at admission (B1) and follow-up (B2)

**A1**

**A2**

**B1**

**B2**

**Appendix 6. Expert-novice interobserver agreement according to the novice sonographers experience acquired throughout the study.**

|  | **Weighted kappa (95%CI)** | |
| --- | --- | --- |
|  | **Admission (aLUS)** | **Follow-up (fLUS)** |
| **All LUS exams** | **N=41** | **N=38** |
| 8-point protocol | 0.54 (0.35 to 0.74) | 0.62 (0.47 to 0.77) |
| 28-point protocol | 0.51 (0.31 to 0.71) | 0.41 (0.25 to 0.57) |
| **First five LUS exams** | **N=28** | **N=32** |
| 8-point protocol | 0.62 (0.39 to 0.84) | 0.65 (0.48 to 0.82) |
| 28-point protocol | 0.52 (0.26 to 0.77) | 0.35 (0.14 to 0.55) |
| **From the sixth LUS exam** | **N=13** | **N=6** |
| 8-point protocol | 0.41 (0.05 to 0.77) | 0.53 (0.27 to 0.78) |
| 28-point protocol | 0.61 (0.30 to 0.92) | 0.53 (0.33 to 0.73) |

**Appendix 7. Bland-Altman difference plots for eight and 28-point protocols at admission and at discharge**


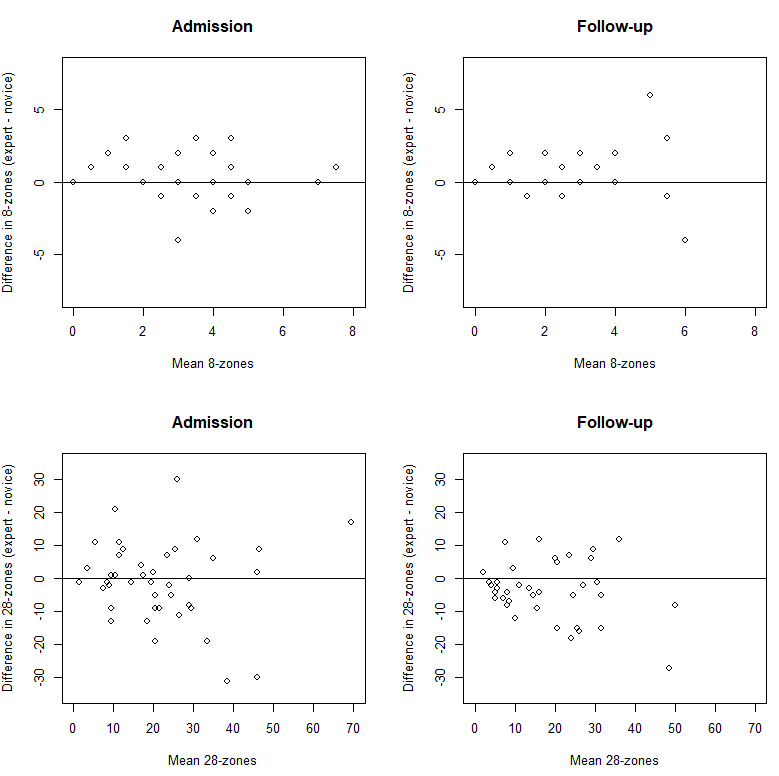


**Eight-point protocol**

Using a t-test for paired data, the mean difference between novices and experts (experts minus novices) was:

0.52 (95%CI 0.06 to 0.99, *P*=0.028) at admission

0.59 (95%CI 0.08 to 1.11, *P*=0.026) at follow-up

**Twenty-eight-point protocol**

Using a t-test for paired data, the mean difference between novices and experts (experts minus novices) was:

-1.1 (95%CI -4.9 to 2.6, *P*=0.55) at admission

-3.5 (95%CI -6.5 to -0.6, *P*=0.020) at follow-up

**Appendix 8. Mean Time for LUS images acquisition and interpretation**

|  | Admisson | | Follow-up | |
| --- | --- | --- | --- | --- |
| **Time for acquisition, mean (SD), min** | Expert | Novice | Expert | Novice |
| 8-point protocol | 2.95 (0.83) | 4.12 (1.35) | 2.80 (0.67) | 4.7 (2.3) |
| 28-point protocol | 6.52 (2.10) | 9.32 (2.60) | 6.23 (2.45) | 9.02 (2.93) |
| Mean time difference (95% CI), min | **-3,6 (-4.2, -3.0)*** | **-5.1 (-5.9, -4.3)*** | **-3.4 (-4.1, -2.8)*** | **-4.3 (-5.1, -3.6)*** |
| **Time for interpretation, mean (SD), min** |  |  |  |  |
| 8-point protocol | 1.23 (0.68) | 1.98 (0.97) | 0.90 (1.0) | 2.23 (1.05) |
| 28-point protocol | 7.22 (2.32) | 8.32 (2.02) | 6.08 (1.87) | 8.20 (3.10) |
| Mean time difference (95% CI), min | **-6.0 (-6.7, -5.3)*** | **-6.3 (-6.9, -5.9)*** | **-5.10 (-5.8, -4.4)*** | **-6.0 (-7.0, -4.9)*** |

*P value <0.001 for all comparison (paired t-test)

**Appendix 9. Correlation between admission-follow-up change in LUS-estimated congestion and body weight, Everest score or NTproBNP evolution.**

##

## **Eight-point protocol**

Dot plots


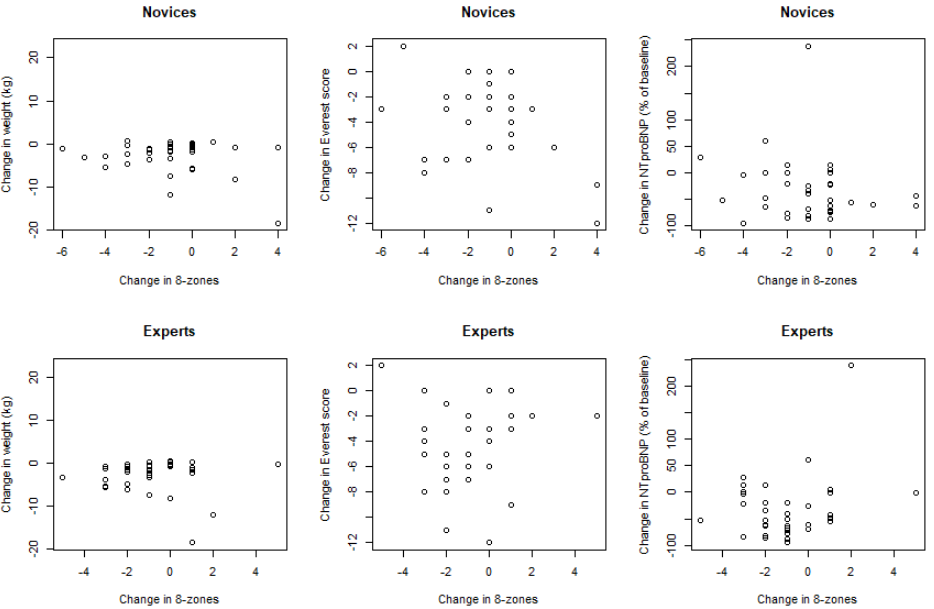


Spearman’s correlation coefficients:

- Novices:
  - Change in weight: ρ= 0.16, p=0.35
  - Change in Everest score: ρ=-0.13, p=0.45
  - Change in NTproBNP (% of baseline): ρ=-0.14, p=0.41
- Experts:
  - Change in weight: ρ=-0.23, p=0.17
  - Change in Everest score: ρ=0.15, p=0.37
  - Change in NTproBNP (% of baseline): ρ=0.05, p=0.77

## **Twenty-eight-point protocol**

Dot plots


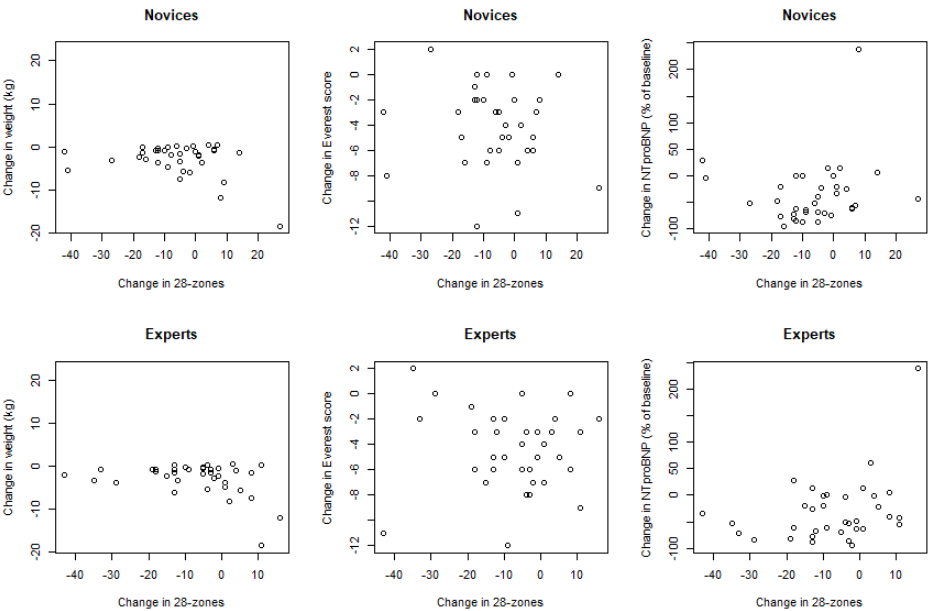


Spearman’s correlation coefficients:

- Novices :
  - Change in weight: ρ=-0.09, p=0.59
  - Change in Everest score: ρ=-0.13, p=0.47
  - Change in NTproBNP (% of baseline): ρ=0.24, p=0.16
- Experts :
  - Change in weight: ρ=-0.21, p=0.20
  - Change in Everest score: ρ=-0.11, p=0.53
  - Change in NTproBNP (% of baseline): ρ=0.28, p=0.09

**Appendix 10. Correlation between admission LUS-estimated congestion and admission Everest score and NT-proBNP.**

**Admission 8-point protocol**

Spearman’s correlation coefficients:

- Novices :
  - Admission Everest score: ρ=0.17, p=0.31
  - Admission NT-proBNP ρ=0.32, p=0.03
- Experts :
  - Admission Everest score: ρ=0.24, p=0.12
  - Admission NT-proBNP: ρ=0.35, p=0.02

**Admission 28-point protocol**

Spearman’s correlation coefficients:

- Novices :
  - Admission Everest score: ρ=0.12, p=0.44
  - Admission NT-proBNP ρ=0.33, p=0.05
- Experts :
  - Admission Everest score: ρ=0.18, p=0.26
  - Admission NT-proBNP ρ=0.42, p=0.005

**Appendix 10. Correlation between follow-up LUS and follow-up Everest score and NT-proBNP.**

**Follow-up 8-point protocol**

Spearman’s correlation coefficients:

- Novices :
  - Follow-up Everest score: ρ=0.24, p=0.16
  - Follow-up NT-proBNP: ρ=0.50, p=0.002
- Experts :
  - Follow-up Everest score: ρ=0.02, p=0.91
  - Follow-up NT-proBNP: ρ=0.50, p=0.002

**Follow-up 28-point protocol**

Spearman’s correlation coefficients:

- Novices :
  - Follow-up Everest score: ρ=0.19, p=0.25
  - Follow-up NT-proBNP: ρ=0.41, p=0.01
- Experts :
  - Follow-up Everest score: ρ=0.28, p=0.09
  - Follow-up NT-proBNP: ρ=0.41, p=0.01

**References**

1. Gheorghiade M, Konstam MA, Burnett JC, Grinfeld L, Maggioni AP, Swedberg K, et al. Short-term clinical effects of tolvaptan, an oral vasopressin antagonist, in patients hospitalized for heart failure - The EVEREST clinical status trials. Jama-J Am Med Assoc. 2007;297(12):1332-43.

2. Gullett J, Donnelly JP, Sinert R, Hosek B, Fuller D, Hill H, et al. Interobserver agreement in the evaluation of B-lines using bedside ultrasound. Journal of Critical Care. 2015;30(6):1395-9.
